# Deep-learning-based image compression for microscopy images: An empirical study

**DOI:** 10.1017/S2633903X24000151

**Published:** 2024-12-20

**Authors:** Yu Zhou, Jan Sollmann, Jianxu Chen

**Affiliations:** 1 Department of Biospectroscopy, Leibniz-Institut für Analytische Wissenschaften – ISAS – e.V., Dortmund, Germany; 2 Faculty of Computer Science, Ruhr University Bochum, Bochum, Germany

**Keywords:** compression, deep learning, in-silico labelling, microscopic images

## Abstract

With the fast development of modern microscopes and bioimaging techniques, an unprecedentedly large amount of imaging data is being generated, stored, analyzed, and shared through networks. The size of the data poses great challenges for current data infrastructure. One common way to reduce the data size is by image compression. This study analyzes multiple classic and deep-learning-based image compression methods, as well as an empirical study on their impact on downstream deep-learning-based image processing models. We used deep-learning-based label-free prediction models (i.e., predicting fluorescent images from bright-field images) as an example downstream task for the comparison and analysis of the impact of image compression. Different compression techniques are compared in compression ratio, image similarity, and, most importantly, the prediction accuracy of label-free models on original and compressed images. We found that artificial intelligence (AI)-based compression techniques largely outperform the classic ones with minimal influence on the downstream 2D label-free tasks. In the end, we hope this study could shed light on the potential of deep-learning-based image compression and raise the awareness of the potential impacts of image compression on downstream deep-learning models for analysis.

## Impact Statement

This empirical study delves into the pressing challenge posed by the escalating amount of biological microscopy imaging data and the consequential strain on existing data infrastructure. Effective image compression methods could help reduce the data size significantly without losing necessary information and therefore reduce the burden on data management infrastructure and permit fast transmission through the network for data sharing or cloud computing. In response, we investigate both classic and deep-learning-based image compression methods within the domain of 2D/3D grayscale bright-field microscopy images and their influence on the downstream task. Our findings unveil the superiority of deep-learning-based techniques, presenting elevated compression ratios while preserving reconstruction quality and with little effect on the downstream data analysis. Hence, the integration of deep-learning-based compression techniques into the existing bioimage analysis pipeline would be immensely beneficial in data sharing and storage.

## 1. Introduction

Image compression is the process of reducing the size of digital images while retaining the useful information for reconstruction. This is achieved by removing redundancies in the image data, resulting in a compressed version of the original image that requires less storage space and can be transmitted more efficiently. In many fields of research, including microscopy, high-resolution images are often acquired and processed, leading to significant challenges in terms of storage and computational resources. In particular, researchers in the microscopy image analysis field are often faced with infrastructure limitations, such as limited storage capacity or network bandwidth. Image compression can help mitigate such challenges, allowing researchers to store and transmit images efficiently without compromising their quality and validity. Lossless image compression refers to the compression techniques preserving every bit of information in data and making error-free reconstruction, ideal for applications where data integrity is paramount. However, the limited size reduction capability, such as a compression ratio of 2 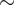 3 as reported by Walker et al.^(^^1^ ^)^, is far from sufficient to alleviate the data explosion crisis. In this work, we focus on lossy compression methods, where some information lost may occur but can yield significantly higher compression ratio.

Image compression has historically been employed to reduce data burdens in various scenarios. For instance, the WebP format is used by web developers to enhance web performance by reducing webpage loading times.^(^^2^ ^)^ Similarly, Apple’s High Efficiency Image File (HEIF) format optimizes storage on mobile devices, improving data transmission and storage efficiency.^(^^3^ ^)^ Despite lossy compression techniques (both classic and deep-learning-based) being widely employed in the computer vision field, their feasibility and impact in the field of biological microscopy images remain largely underexplored.

In this paper, we propose a two-phase evaluation pipeline, compression algorithm comparison and downstream task analysis in the context of microscopy images. To fully explore the impact of lossy image compression on downstream image analysis tasks, we employed a set of label-free models, a.k.a., in-silico labeling.^(^^4^ ^)^ A label-free model denotes a deep-learning approach capable of directly predicting fluorescent images from transmitted light bright-field images.^(^^5^ ^)^ Considering the large amount of bright-field images being used in regular biological studies, it is of great importance that such data compression techniques can be utilized without compromising the prediction quality.

Through intensive experiments, we demonstrated that deep-learning-based compression methods can outperform the classic algorithms in terms of compression ratio, and post-compression reconstruction quality, and their impact on the downstream label-free task, indicating their huge potentials in the bioimaging field. Meanwhile, we made a preliminary attempt to build 3D compression models and reported the current limitation and possible future directions. Overall, we want to raise the awareness of the importance and potentials of deep-learning-based compression techniques and hopefully help in the strategical planning of future data infrastructure for bioimaging.

Specifically, the main contribution of the paper is:

1. Benchmark common classic and deep-learning-based image compression techniques in the context of 2D grayscale bright-field microscopy images.
2. Empirically investigate the impact of data compression to the downstream label-free tasks.
3. Expand the scope of the current compression analysis for 3D microscopy images.

The remaining of this paper is organized as follows: Section 2 will introduce classic and deep-learning-based image compression techniques, followed by the method descriptions in Section 3 and experimental settings in Section 4. Results and discussions will be presented in Section 5 with conclusions in Section 7.

## 2. Related works

The classic data compression techniques have been well studied in the last few decades, with the development of JPEG,^(^^6^ ^)^ a popular lossy compression algorithm since 1992, and its successors, JPEG 2000,^(^^7^ ^)^ JPEG XR,^(^^8^ ^)^ and so forth. In recent years, some more powerful algorithms, such as limited error raster compression (LERC), are proposed. Generally, the compression process approximately involves the following steps: color transform (with optional downsampling), domain transform (e.g., discrete cosine transform^(^^9^ ^)^ in JPEG), quantization, and further lossless entropy coding (e.g., run-length encoding or Huffmann coding^(^^10^ ^)^).

**Figure 1.**
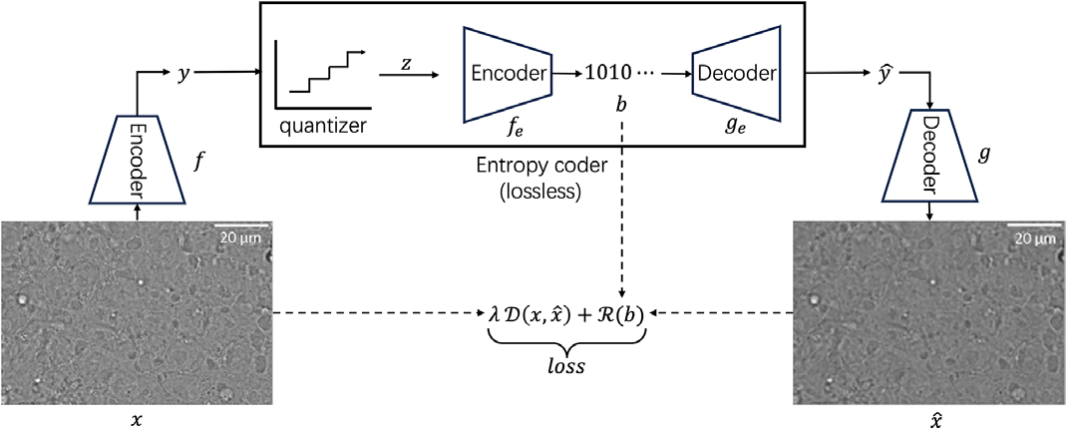
The workflow of a typical learning-based lossy image compression. The raw image 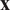 is fed into the encoder 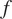 and obtain the low-dimensional latent representation 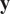 . Then, the lossless entropy coder can further exploit the information redundency: 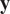 will be firstly quantized to 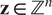 , and then compressed to the bitstream 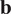 by the entropy encoder 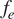 . This bitstream can be stored for transmission or further decompression. The corresponding entropy decoder 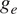 is responsible for the decompression and yield the reconstructed latent representation 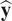 . Lastly, 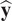 is transmitted to the neural decoder 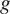 , yielding the reconstructed image 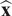 . The loss function of the system is composed of 2 parts: distortion 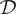 and rate 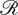 . Distortion represents the reconstruction quality (e.g., Structural Similarity Index Measure [SSIM] between 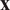 and 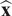 ) while rate focuses more on the compression ability. 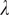 acts as the hyper-parameter to balance the rate–distortion trade-off.

Recently, deep-learning-based image compression gained popularity thanks to the significantly improved compression performance. Roughly speaking, a deep-learning-based compression model consists of two sub-networks: a neural encoder 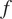 that compresses the image data and a neural decoder 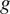 that reconstructs the original image from the compressed representation. Besides, the latent representation will be further losslessly compressed by some entropy coding techniques (e.g., arithmetic coding^(^^11^ ^)^) as seen in Figure 1. Specially, the latent vector will be firstly discretized into 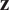 : 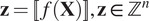 . Afterward, 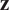 will be encoded/decoded by the entropy coder ( 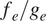 ) and decompressed by the neural decoder 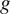 : 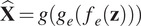 . The objective is to minimize the loss function containing rate–distortion trade-off^(^^12^ ^,^^13^ ^)^:
(1)

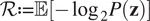



(2)

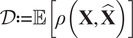



(3)

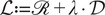

 where 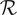 corresponds to the rate loss term, which highlights the compression ability of the system. 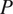 is the entropy model that provides prior probability to the entropy coding, and 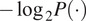 denotes the information entropy and can approximately estimate the optimal compression ability of the entropy encoder 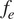 , defined by the Shannon theory.^(^^13^ ^,^ ^14^ ^)^ 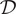 is the distortion term, which can control the reconstruction quality. 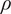 is the norm or perceptual metric, for example, MSE, MS-SSIM,^(^^15^ ^)^ and so forth. The trade-off between these two terms is achieved by the scale hyper-parameter 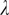 .

Because the lossless entropy coding entails the accurate modeling of the prior probability of the quantized latent representation 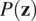 , Ballé et al.^(^^16^ ^)^ justified that there exist statistical dependencies in the latent representation using the current fully-factorized entropy model, which will lead to suboptimal performance and not be adaptive to all images. To further improve the entropy model, Ballé et al. propose a *hyperprior* approach,^(^^16^ ^)^ where a hyper latent 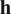 (also called *side information*) is generated by the auxillary neural encoder 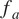 from the latent space 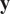 : 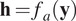 , then the scale parameter of the entropy model can be estimated by the output of the auxillary decoder 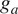 : 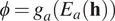 so that the entropy model can be adaptively adjusted by the input image 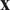 , with the bit-rate further enhanced. Minnen et al.^(^^17^ ^)^ extended the work to get the more reliable entropy model by jointly combining the data from the above mentioned *hyperprior* and the proposed autoregressive *Context Model.*

Besides the improvement in the entropy model, lots of effort is also put into the enhancement of the network architecture. Ballé et al.^(^^18^ ^)^ replaced the normal RELU activation with the proposed generalized division normalization (GDN) module to better capture the image statistics. Johnston et al.^(^^19^ ^)^ optimized the GDN module in a computationally efficient manner without sacrificing the accuracy. Cheng et al.^(^^20^ ^)^ introduced the skip connection and attention mechanism. The transformer-based auto-encoder was also reported for data compression in recent years.^(^^21^ ^)^

## 3. Methodology

**Figure 2.**
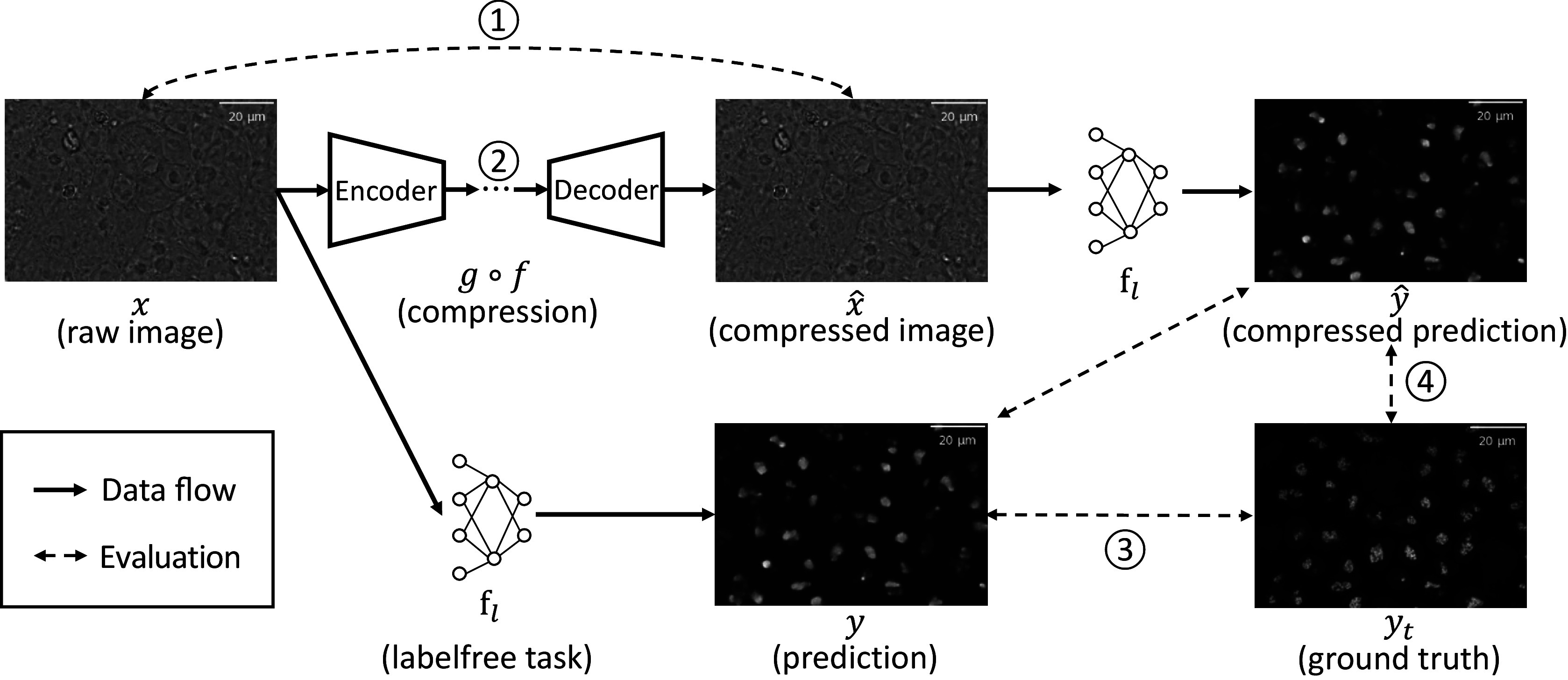
Overview of our proposed evaluation pipeline. The objective is to fully estimate the compression performance of different compression algorithms (denoted as 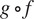 ) in the bioimage field and investigate their influence to the downstream AI-based bioimage analysis tasks (e.g., label-free task in this study, denoted as 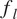 ). The solid line represents data flow while the dash line means evaluation. The bright-field raw image 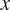 will be compressed and decompressed: 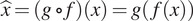 . Then, we feed the reconstructed 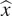 to the label-free model 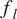 to get the estimated fluorescent image 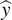 : 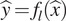 . Meanwhile, normal prediction 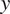 is also made by 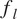 from the raw image 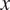 : 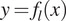 . Regarding the evaluation, ①\② exhibits the rate–distortion ability of the compression algorithm, ③\④\⑤ represents their influence to the downstream task 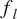 . Specifically, ① measures the reconstruction ability of the compression method while ② records the bit-rate and can reflect the compression ratio ability. ③ and ④ represents the prediction accuracy of the 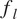 model using the raw image 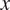 and the reconstructed image 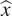 as input, respectively. ⑤ measures the similarity between these two predictions.

The evaluation pipeline was proposed in this study to benchmark the performance of the compression model in the bioimage field and estimate their influence to the downstream label-free generation task. As illustrated in Figure 2, the whole pipeline contains two parts: compression part: 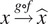 and downstream label-free part: 
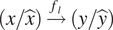
 , where the former is designed to measure the rate–distortion performance of the compression algorithms and the latter aims to quantify their influence to the downstream task.

During the compression part, the raw image 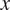 will be transformed to the reconstructed image 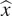 through the compression algorithm 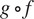 :
(4)

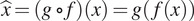

where 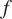 represents the compression process, and 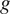 denotes the decompression process. Note that the compression methods could be both classic strategies (e.g., JPEG) and deep-learning-based algorithms. The performance of the algorithm can be evaluated through rate–distortion performance, as explained in (1) to (3).

In the downstream label-free part, the prediction will be made by the model 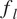 using both the raw image 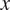 and the reconstructed image 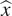 :
(5)

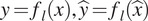



The evaluation to measure the compression influence to the downstream tasks is made by:
(6)

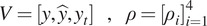



(7)

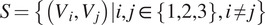



(8)

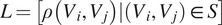

 where the evaluation metric L is the collection of different metrics 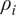 on different image pairs 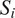 . V is the collection of the raw prediction 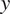 , prediction made by the reconstructed image 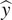 and the ground truth 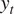 . S is formed by pairwise combinations of elements from V. 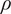 represents the metric we used to measure the relation between image pairs. In this study, we totally utilized four metrics: learned perceptual image patch similarity (LPIPS),^(^^22^ ^)^ SSIM, peak signal-to-noise ratio (PSNR), and Pearson correlation.

To conclude, through the above proposed two-phase evaluation pipeline, the compression performance of the compression algorithm will be fully estimated, and their impact on the downstream task will also be well investigated.

## 4. Experimental settings

### 4.1. Dataset

The dataset used in this study is the human-induced pluripotent stem cell single-cell image dataset^(^^23^ ^)^ released by the Allen Institute for Cell Science. We utilized grayscale bright-field images and its corresponding fluorescent image pairs from the fibrillarin cell line, where the dense fibrillar component of the nucleolus is endogenously tagged. For 3D experiments, 500 samples were chosen from the dataset, with 395 for training and the remaining 105 samples for evaluation. While in terms of 2D experiments, the middle slice of each 3D sample was extracted, resulting in 2D slices of 624 × 924 pixels.

### 4.2. Implementation details

During the first compression part of the proposed two-phase evaluation pipeline, we made the comparison using both classic methods and deep-learning-based algorithms. In terms of the classic compression, we employed the Python package “tifffile” to apply 3 classic image compression: JPEG 2000, JPEG XR, and LERC, focusing on level 8 for the highest image quality preservation. To enhance compression efficiency, we used a 16 × 16-pixel tile-based approach, facilitating image data access during compression and decompression. This methodology enabled a thorough exploration of the storage versus image quality trade-off.

Regarding learning-based methods, 6 pre-trained models proposed in refs. (3, 17, 20) were applied in 2D compression, with each kind of model trained with 2 different metrics (MSE and MS-SSIM), resulting in 12 models in total. The pretrained checkpoints were provided by the CompressAI tool.^(^^24^ ^)^ For the 3D senario, an adapted bmshj2018-factorized compression model^(^^16^ ^)^ was trained and evaluated on our microscopy dataset. For the first 50 epochs, MSE metric was employed in the reconstruction loss term, followed by MS-SSIM metric for another 50 epochs to enhance the image quality.

When it comes to the second label-free generation part, the pretrained Pix2Pix 2D (Fnet 2D as the generator) and Fnet 3D model were obtained from the mmv_im2im Python package.^(^^25^ ^)^ All the label-free 2D/3D models were trained by raw images. Detailed training recipes are listed in Supplementary Tables S3 and S4.

## 5. Results

In this section, we will present and analyze the performance of the image compression algorithms and their impact on the downstream label-free task, using the proposed two-phase evaluation pipeline.

### 5.1. Data compression results

First, we did the compression performance comparison experiment in the context of grayscale microscopic bright-field image, based on the first part of the evaluation pipeline. The results show that deep-learning-based compression algorithms behave well in terms of the reconstruction quality and compression ratio ability in both 2D and 3D cases and outperform the classic methods.

The second to the fourth rows in Table 1 and Supplementary Table S1 demonstrate the quantitative rate–distortion performance for the three traditional compression techniques involved. Although the classic method LERC achieved the highest result in all the quality metrics for the reconstructed image, it just saves 12.36% of the space, which is way lower compared to the deep-learning-based methods. Meanwhile, JPEG-2000-LOSSY can achieve comparable compression ratios with respect to AI-based algorithms, but its quality metric ranks the bottom, with only 0.158 in correlation and 0.424 in SSIM. The above results compellingly showcase that the classic methods cannot make a trade-off in the rate–distortion performance.

**Table 1.** Evaluation of the average 2D bright-field image quality for the different compression methods compared to the original image, to test the reconstruction ability

Compression	LPIPS	SSIM	Correlation	PSNR ( 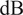 )
Original	0	1	1	108.1308
JPEGXR	0.273 ± 0.060	0.828 ± 0.059	0.899 ± 0.048	30.499 ± 2.780
JPEG–2000-LOSSY	0.599 ± 0.069	0.424 ± 0.104	0.158 ± 0.213	15.852 ± 4.846
LERC	**0.020** ± 0.034	**0.980** ± 0.036	**0.993** ± 0.020	**51.720** ± 21.571
bmshj2018-factorized-mse–8	0.198 ± 0.072	0.962 ± 0.019	0.984 ± 0.008	**38.474** ± 3.059
bmshj2018-factorized-ms-ssim–8	0.163 ± 0.053	0.970 ± 0.015	0.986 ± 0.009	37.370 ± 2.642
bmshj2018-hyperprior-mse–8	0.207 ± 0.074	0.959 ± 0.023	0.983 ± 0.010	38.436 ± 3.024
bmshj2018-hyperprior-ms-ssim–8	0.168 ± 0.057	0.969 ± 0.016	0.985 ± 0.008	37.171 ± 2.631
mbt2018-mean-mse–8	0.217 ± 0.078	0.956 ± 0.023	0.982 ± 0.010	37.975 ± 3.101
mbt2018-mean-ms-ssim–8	0.171 ± 0.059	0.970 ± 0.015	**0.987** ± 0.008	37.672 ± 2.681
mbt2018-mse–8	0.206 ± 0.076	0.956 ± 0.023	0.982 ± 0.010	38.169 ± 3.030
mbt2018-ms-ssim–8	**0.162** ± 0.056	**0.971** ± 0.015	0.986 ± 0.008	37.287 ± 2.566
cheng2020-anchor-mse–6	0.280 ± 0.100	0.913 ± 0.035	0.961 ± 0.016	34.373 ± 2.654
cheng2020-anchor-ms-ssim–6	0.207 ± 0.071	0.954 ± 0.018	0.974 ± 0.009	33.425 ± 2.798
cheng2020-attn-mse–6	0.275 ± 0.097	0.914 ± 0.035	0.961 ± 0.015	34.561 ± 2.685
cheng2020-attn-ms-ssim–6	0.204 ± 0.070	0.954 ± 0.018	0.973 ± 0.009	34.043 ± 2.746

First column: compression methods, with the second to the fourth rows as the classic methods and fifth to the last as the deep-learning-based methods. The second to the last columns indicate the four metrics that we used to measure the reconstruction ability: LPIPS (the smaller the better), SSIM, Correlation, PSNR (the larger the better).

**Figure 3.**
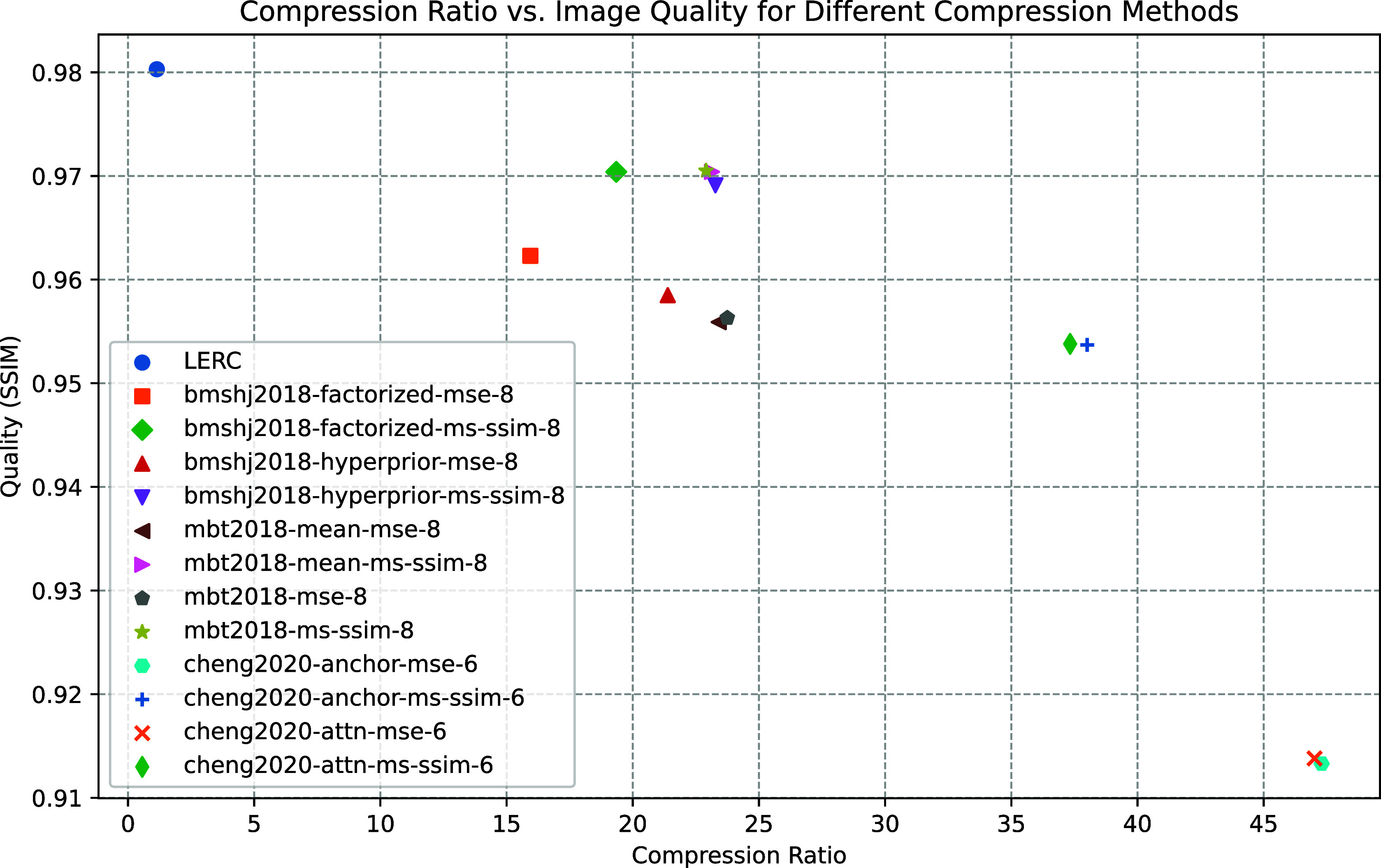
Compression ratio versus image reconstruction quality (SSIM) for different compression methods. It is evident that there is a trade-off between the compression ratio and the image reconstruction quality. Note that JPEGXR and JPEG-2000-LOSSY are invisible due to the low quality.

**Figure 4.**
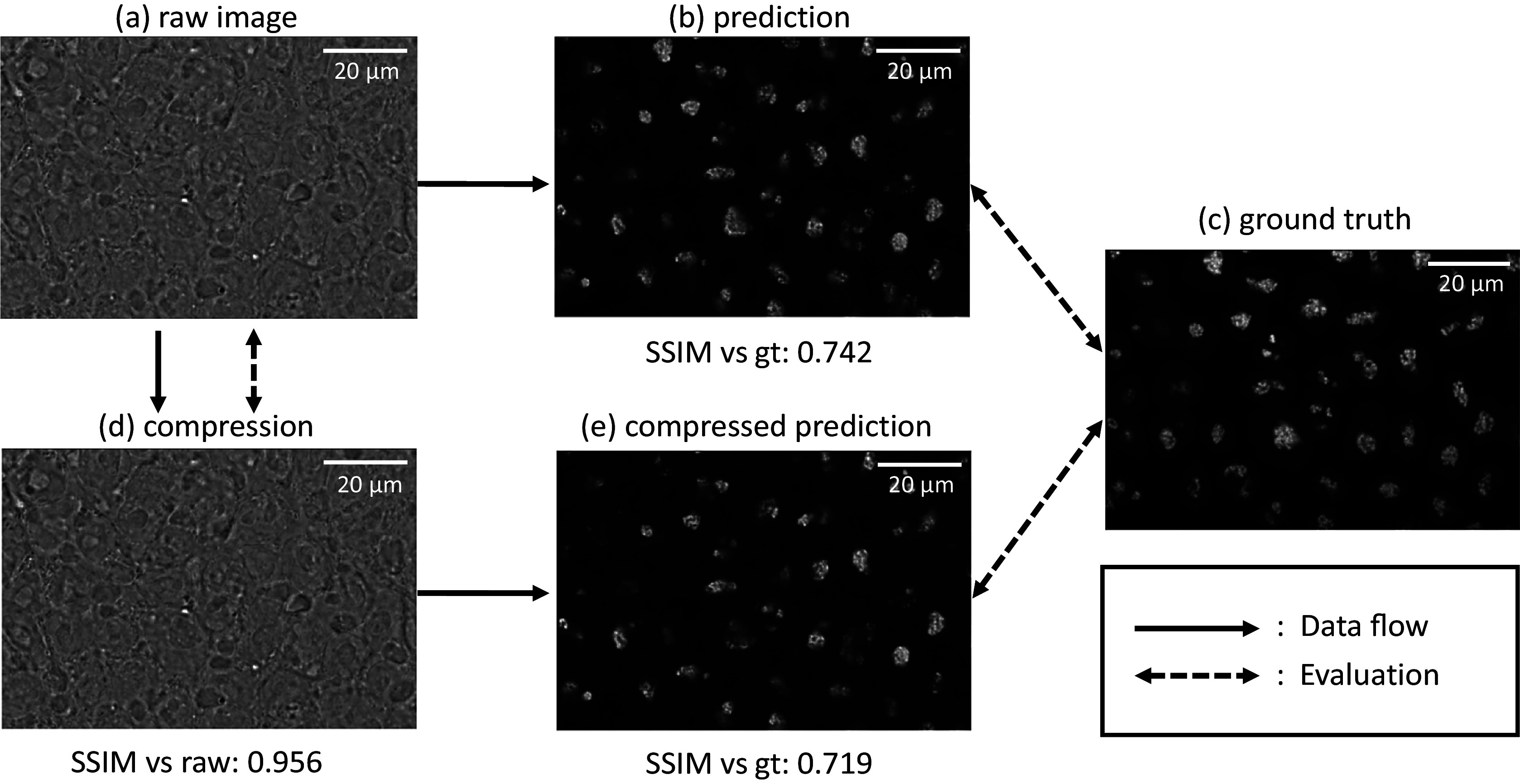
Visualization of 2D bright-field image compression result (first row, model: mbt2018 (mse)) + downstream label-free model prediction (second row). The upper right compression result is visually plausible compared to the input, and the compressed prediction (bottom left) using the label-free model is very close to the original prediction (bottom middle), which suggests the minimal influence of the selected deep-learning-based compression to the downstream task.

Besides, results from deep-learning models exhibit close similarities, yielding favorable outcomes, as illustrated in Table 1 and Supplementary Table S1 from the fifth row to the last. From Figure 3, it is evident that there is a trade-off between the image quality and the compression ratio. Notably, the “mbt2018-ms-ssim-8” method exhibits a slight advantage in terms of SSIM, achieving a value of 0.971. Conversely, the “mbt2018-mean-ms-ssim-8” method showcases a slight edge in correlation, with a score of 0.987. When considering compression ratio, “cheng2020-anchor-mse-6” outperforms the others, with an compression ratio of 47.298. A sample result is visualized in Figure 4.

**Figure 5.**
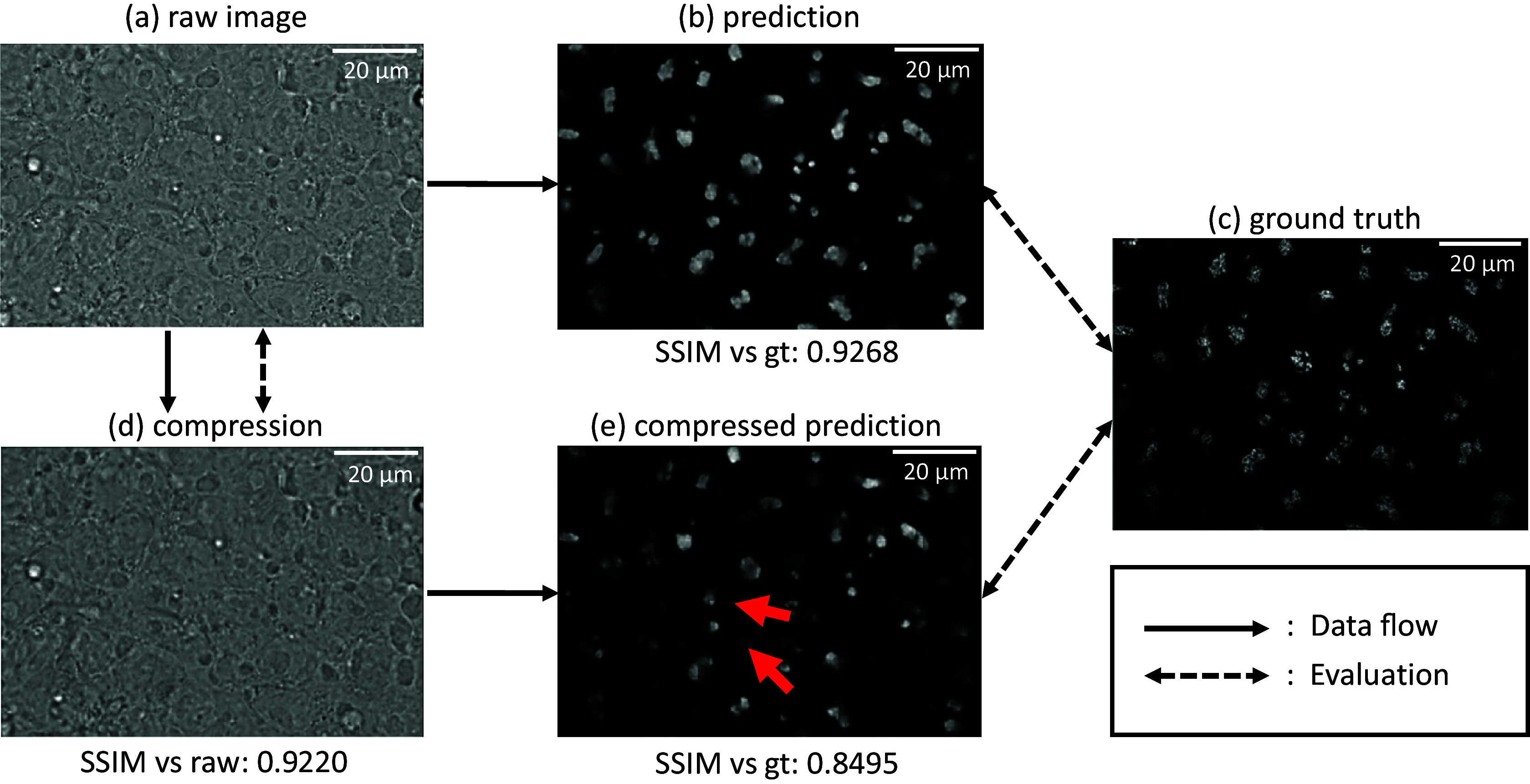
Visualization of 3D compression result based on the bmshj2018-factorized model.

As illustrated in Figure 5, the 3D compression result is visually plausible and the quantitative evaluation metrics are listed in the first row in Table 4. The metrics are relatively high, reaching 0.922 in SSIM and 0.949 in correlation. Regarding the compression ratio, 97.74 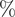 of space will be saved.

In brief, the above findings suggest that deep-learning-based compression methods behave well in the context of microscopic image field and averagely outperform the classic methods in terms of reconstruction ability and compression ratios.

### 5.2. Downstream label-free results

We also conducted an experiment to assess the impact of the aforementioned compression techniques on downstream AI-based bioimage analysis tasks, specifically the label-free task in our study (please refer to the Supplementary Case Study section for the analysis of additional downstream tasks). Our results indicate that in 2D cases, the prediction accuracy is higher when the input image is compressed using deep-learning-based methods, as opposed to traditional methods. Furthermore, this accuracy closely aligns with the predictions derived from the raw image, suggesting that deep-learning-based compression methods have a minimal impact on the downstream task.

Tables 2 and 3 exhibit the influence of data compression to the downstream label-free task in 2D cases. Regarding the comparison of the accuracy between the predictions using compressed input and original input (Table 2), we found that although the slight degradation in correlation and PSNR, the average SSIM value among deep-learning-based methods is akin to the original prediction and surpasses the classic methods, with “bmshj2018-hyperprior-ms-ssim-8” model reaching the highest value (0.752). If we compare the similarity between the predictions using compressed images and original images (Table 3), “mbt2018-ms-ssim-8” and LERC ranked the highest in SSIM and correlation, respectively.

**Table 2.** Evaluation of the average prediction quality for the different compression methods compared to the ground truth, to test the impact of the compression methods to the label-free task

Compression	LPIPS	SSIM	Correlation	PSNR ( 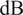 )
Original	0.134 ± 0.033	0.742 ± 0.073	0.736 ± 0.102	24.542 ± 1.443
JPEGXR	0.200 ± 0.052	0.656 ± 0.111	0.404 ± 0.138	22.852 ± 1.814
JPEG–2000-LOSSY	0.523 ± 0.149	0.216 ± 0.183	0.001 ± 0.037	13.571 ± 5.756
LERC	0.145 ± 0.038	0.653 ± 0.167	0.735 ± 0.102	23.351 ± 2.615
bmshj2018-factorized-mse–8	0.148 ± 0.040	0.735 ± 0.082	0.606 ± 0.136	24.228 ± 1.732
bmshj2018-factorized-ms-ssim–8	0.135 ± 0.031	0.744 ± 0.075	0.705 ± 0.099	24.713 ± 1.466
bmshj2018-hyperprior-mse–8	0.164 ± 0.057	0.703 ± 0.115	0.564 ± 0.149	23.735 ± 2.087
bmshj2018-hyperprior-ms-ssim–8	0.135 ± 0.029	**0.752** ± 0.071	0.682 ± 0.105	24.710 ± 1.612
mbt2018-mean-mse–8	0.157 ± 0.042	0.728 ± 0.090	0.567 ± 0.148	24.007 ± 1.735
mbt2018-mean-ms-ssim–8	**0.134** ± 0.030	0.751 ± 0.072	0.703 ± 0.100	24.744 ± 1.577
mbt2018-mse–8	0.156 ± 0.043	0.719 ± 0.096	0.581 ± 0.141	23.995 ± 1.650
mbt2018-ms-ssim–8	0.135 ± 0.030	0.747 ± 0.073	**0.707** ± 0.096	**24.746** ± 1.514
cheng2020-anchor-mse–6	0.266 ± 0.114	0.519 ± 0.220	0.357 ± 0.184	20.958 ± 3.581
cheng2020-anchor-ms-ssim–6	0.154 ± 0.034	0.717 ± 0.084	0.626 ± 0.109	24.247 ± 1.619
cheng2020-attn-mse–6	0.276 ± 0.121	0.507 ± 0.225	0.331 ± 0.188	20.624 ± 3.704
cheng2020-attn-ms-ssim–6	0.149 ± 0.032	0.734 ± 0.076	0.624 ± 0.114	24.351 ± 1.693

First column: compression methods, with the second to the fourth rows as the classic methods and fifth to the last as the deep-learning-based methods.

**Table 3.** Evaluation of the average prediction quality for the different compression methods compared to the original prediction

Compression	LPIPS	SSIM	Correlation	PSNR ( 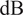 )
Original	0.000 ± 0.000	1.000 ± 0.000	1.000 ± 0.000	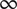
JPEGXR	0.172 ± 0.042	0.791 ± 0.085	0.512 ± 0.158	23.232 ± 1.772
JPEG–2000-LOSSY	0.500 ± 0.138	0.294 ± 0.214	0.005 ± 0.042	13.893 ± 5.632
LERC	0.013 ± 0.026	0.915 ± 0.156	0.999 ± 0.002	49.658 ± 15.585
bmshj2018-factorized-mse–8	0.097 ± 0.048	0.888 ± 0.070	0.791 ± 0.150	27.195 ± 3.325
bmshj2018-factorized-ms-ssim–8	0.053 ± 0.017	0.936 ± 0.028	**0.937** ± 0.036	31.277 ± 2.080
bmshj2018-hyperprior-mse–8	0.119 ± 0.063	0.854 ± 0.110	0.730 ± 0.181	25.977 ± 3.537
bmshj2018-hyperprior-ms-ssim–8	0.065 ± 0.025	0.925 ± 0.033	0.902 ± 0.064	29.753 ± 2.607
mbt2018-mean-mse–8	0.113 ± 0.053	0.870 ± 0.076	0.733 ± 0.178	26.270 ± 3.368
mbt2018-mean-ms-ssim–8	0.054 ± 0.019	0.937 ± 0.026	0.933 ± 0.042	31.225 ± 2.313
mbt2018-mse–8	0.108 ± 0.051	0.871 ± 0.076	0.752 ± 0.164	26.556 ± 3.487
mbt2018-ms-ssim–8	**0.052** ± 0.019	**0.939** ± 0.028	**0.937** ± 0.040	**31.465** ± 2.373
cheng2020-anchor-mse–6	0.230 ± 0.105	0.653 ± 0.222	0.444 ± 0.239	21.375 ± 3.589
cheng2020-anchor-ms-ssim–6	0.097 ± 0.031	0.879 ± 0.047	0.808 ± 0.101	26.772 ± 2.160
cheng2020-attn-mse–6	0.240 ± 0.111	0.639 ± 0.231	0.413 ± 0.245	21.043 ± 3.744
cheng2020-attn-ms-ssim–6	0.094 ± 0.031	0.887 ± 0.042	0.809 ± 0.105	26.910 ± 2.303

First column: compression methods, with the second to the fourth rows as the classic methods and fifth to the last as the deep-learning-based methods.

When it comes to 3D cases, the prediction from the compressed image is not comparable to that predicted by the raw bright-field image (2.54 dB 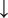 in PSNR and 0.08 dB 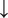 in SSIM), as shown in the second and third rows from Table 4, indicating a quality downgrade during compression. This can be attributed primarily to the ignorance of considering compression in the training phase of the label-free model. Notably, the accuracy gap is mitigated when the label-free model is also trained with the compressed images. As illustrated in Figure 5, despite the visually plausible reconstruction result, the information loss during the compression process also heavily affects the downstream label-free generation task. For instance, the fibrillarin structure pointed by the arrow in the prediction result from the compressed image is missing, which is quite obvious in the corresponding prediction from the raw image.

**Table 4.** 3D compression results using the bmshj2018-factorized model

Comparison	LPIPS	SSIM	Correlation	**PSNR** ( 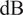 )
Raw image vs compressed image	0.251 ± 0.056	0.922 ± 0.024	0.948 ± 0.015	28.137 ± 6.977
Prediction vs gt	0.405 ± 0.043	0.927 ± 0.030	0.907 ± 0.022	32.606 ± 1.840
Compressed prediction vs gt	0.486 ± 0.038	0.850 ± 0.066	0.598 ± 0.128	30.061 ± 1.306
compressed prediction vs prediction	0.259 ± 0.036	0.820 ± 0.077	0.658 ± 0.129	28.543 ± 1.569
Compressed prediction (with compressed training) vs gt	0.487 ± 0.042	0.895 ± 0.035	0.853 ± 0.037	28.261 ± 1.131

The table evaluates both compression performance (first row) and its impact on downstream tasks (rows 2–4). In addition, it compares results from compressed training (fifth row).

Briefly, the above result suggests that in 2D cases, the downstream task will be less affected when deep-learning-based methods were applied. However, the prediction accuracy will be largely affected in 3D cases.

### 5.3. Label-free results with compressed training

**Figure 6.**
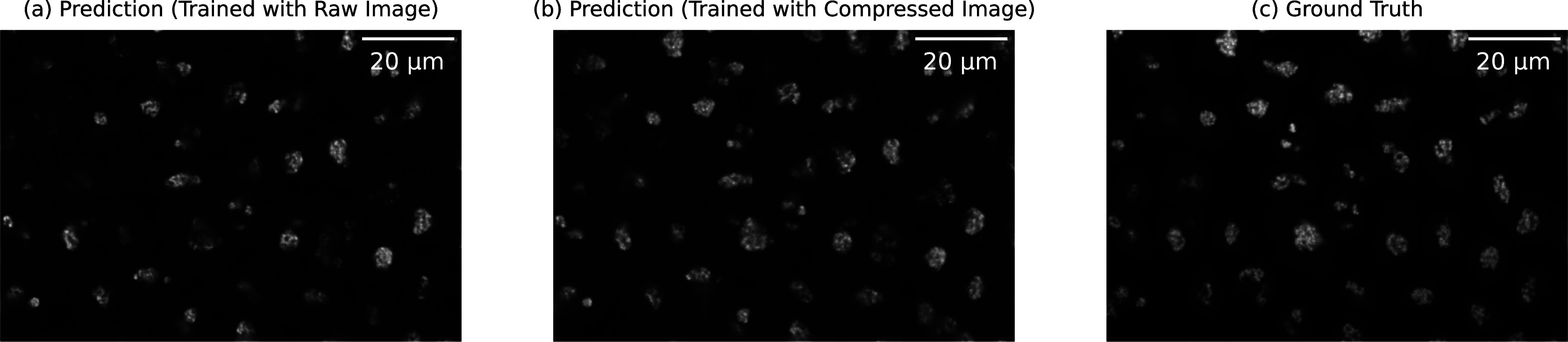
The prediction result of the downstream label-free models trained with raw/lossy compressed images, respectively. The input is the lossy compressed bright-field images using mbt2018 (mse) model. (a) Prediction from a label-free model trained with raw uncompressed images,^(^^26^ ^)^ (b) Prediction from a label-free model trained with images compressed with mbt2018 (mse) model, (c) The ground truth. The label-free model trained on uncompressed data fails to produce accurate results when applied to lossy compressed images, as evidenced by the visible artifacts. This highlights the incompatibility between the model trained on original data and the application of lossy compression.

Given that the 2D label-free models were all trained with raw uncompressed images, it is also crucial to measure the impact of compression during the training phase in the downstream label-free task. For this purpose, we devised the following experiment: Two label-free models were trained with raw uncompressed data and data compressed using *mbt2018* (mse) model, respectively. Therefore, we compared the performance of these models on the test images also compressed using *mbt2018* (mse) model. As illustrated in Figure 6, we observed significant artifacts in the prediction when the model was not trained on the compressed data used as input, which is subject to the relative low quality metrics shown in Table 2. However, artifacts were almost mitigated when the model was trained with data using the same compression algorithm, which has the closer data distribution. A similar phenomenon is observed in other AI-based compression scenarios (see Supplementary Table S2), where correlation improves when the label-free model is trained with compressed data. The above phenomenon highlights the importance of considering compression in the training process in order to achieve favorable outcomes.

## 6. Discussion

The AI-based compression method used in the proposed evaluation pipeline has several shortcomings. First, in 2D cases, only pre-trained models are used. It would perform better if we fine-tuned the compression model on the microscopy dataset. In addition, to achieve optimal downstream task performance, the model for the downstream task should also be trained with the compressed data. This requirement restricts its application if the model was already trained beforehand, which is often the case. Furthermore, the encoding and decoding latency is higher compared to traditional compression methods.

Regardless of these drawbacks, the potential for integrating image compression with current data guidelines, while emphasizing the preservation of original data, is promising. Bioimage storage platforms could leverage this approach by enabling users to download compressed latent representations for quick preview and assessment using offline decoder. This strategy allows biologists to efficiently screen large datasets, conserving storage and bandwidth. Subsequently, researchers can access the original high-resolution data for in-depth analysis when needed.

## 7. Conclusion

In this research, we proposed a two-phase evaluation pipeline to benchmark the rate–distortion performance of different data compression techniques in the context of grayscale microscopic brightfield images and fully explored the influence of such compression on the downstream label-free task. We found that AI-based image compression methods can significantly outperform classic compression methods and have minor influence on the following label-free model prediction. Despite some limitations, we hope that our work can raise the awareness of the application of deep-learning-based image compression in the bioimaging field and provide insights into the way of integration with other AI-based image analysis tasks.

## Supporting information

Zhou et al. supplementary material Zhou et al. supplementary material

## Data Availability

The codebase has been released at https://github.com/MMV-Lab/data-compression. The data are from the public hiPSC single cell image dataset from the Allen Institute for Cell Science: https://open.quiltdata.com/b/allencell/packages/aics/hipsc_single_cell_image_dataset. The checkpoints and configs are available at https://zenodo.org/records/13134355.

## Supplementary material

The supplementary material for this article can be found at http://doi.org/10.1017/S2633903X24000151.

## References

[1] Walker LA, Li Y, Mcglothlin M and Cai D (2023) A comparison of lossless compression methods in microscopy data storage applications: microscopy compression comparison. In Proceedings of the 2023 6th International Conference on Software Engineering and Information Management. ACM, pp. 154–159.

[2] Ginesu G, Pintus M and Giusto DD (2012) Objective assessment of the webp image coding algorithm. Signal Processing: Image Communication 27(8), 867–874.

[3] Lainema J, Hannuksela MM, Vadakital VKM and Aksu EB (2016) Hevc still image coding and high efficiency image file format. In 2016 IEEE International Conference on Image Processing (ICIP). IEEE, pp 71–75.

[4] Christiansen EM, Yang SJ, Ando DM, Javaherian A, Skibinski G, Lipnick S, Mount E, O’Neil A, Shah K, Lee AK (2018) In silico labeling: predicting fluorescent labels in unlabeled images. Cell 173(3), 792–803. 29656897 10.1016/j.cell.2018.03.040 PMC6309178

[5] Ounkomol C, Seshamani S, Maleckar MM, Collman F and Johnson GR (2018) Label-free prediction of three-dimensional fluorescence images from transmitted-light microscopy. Nature Methods 15(11), 917–920. 30224672 10.1038/s41592-018-0111-2 PMC6212323

[6] Wallace G (1992) The jpeg still picture compression standard. IEEE Transactions on Consumer Electronics 38(1), xviii–xxxiv.

[7] Marcellin MW, Gormish MJ, Bilgin A and Boliek MP (2000) An overview of jpeg-2000. In Proceedings DCC 2000. Data Compression Conference. IEEE, pp. 523–541.

[8] Dufaux F, Sullivan GJ and Ebrahimi T (2009) The JPEG XR image coding standard standards in a nutshell. IEEE Signal Processing Magazine 26(6), 195–204.

[9] Ahmed N, Natarajan T and Rao KR (1974) Discrete cosine transform. IEEE Transactions on Computers 100(1), 90–93.

[10] Huffman DA (1952) A method for the construction of minimum-redundancy codes. Proceedings of the IRE 40(9), 1098–1101.

[11] Rissanen J & Langdon GG (1979) Arithmetic coding. IBM Journal of Research and Development 23(2), 149–162.

[12] Cover TM (1999) Elements of Information Theory. John Wiley & Sons.

[13] Shannon CE (1959) Coding theorems for a discrete source with a fidelity criterion. IRE National Convention Record 4(142–163), 1.

[14] Shannon CE (1948) A mathematical theory of communication. The Bell System Technical Journal 27(3), 379–423.

[15] Wang Z, Simoncelli EP and Bovik AC (2003) Multiscale structural similarity for image quality assessment. In The Thrity-Seventh Asilomar Conference on Signals, Systems & Computers, 2003. IEEE, Vol. 2, pp. 1398–1402.

[16] Ballé J, Minnen D, Singh S, Hwang SJ and Johnston N (2018) Variational image compression with a scale hyperprior. In 6th International Conference on Learning Representations, ICLR 2018 - Conference Track Proceedings. Published by OpenReview.net.

[17] Minnen D, Ballé J and Toderici GD (2018) Joint autoregressive and hierarchical priors for learned image compression. Advances in Neural Information Processing Systems 31, 10771–10780.

[18] Ballé J, Laparra V and Simoncelli EP (2015) Density modeling of images using a generalized normalization transformation. arXiv; *preprint arXiv:1511.06281.* 10.48550/arXiv.1511.06281.

[19] Johnston N, Eban E, Gordon A and Ballé J (2019) Computationally efficient neural image compression. arXiv; *preprint arXiv:1912.08771.* 10.48550/arXiv.1912.08771.

[20] Cheng Z, Sun H, Takeuchi M and Katto J (2020) Learned image compression with discretized Gaussian mixture likelihoods and attention modules. In Proceedings of the IEEE Computer Society Conference on Computer Vision and Pattern Recognition. IEEE, pp. 7936–7945.

[21] Zhu Y, Yang Y and Cohen T (2021) Transformer-based transform coding. In International Conference on Learning Representations. Published by OpenReview.net.

[22] Zhang R, Isola P, Efros AA, Shechtman E and Wang O (2018) The unreasonable effectiveness of deep features as a perceptual metric. In Proceedings of the IEEE Conference on Computer Vision and Pattern Recognition. IEEE, pp. 586–595.

[23] Viana MP, Chen J, Knijnenburg TA, Vasan R, Yan C, Arakaki JE, Bailey M, Berry B, Borensztejn A and Brown EM (2023) Integrated intracellular organization and its variations in human ips cells. Nature 613(7943), 345–354. 36599983 10.1038/s41586-022-05563-7 PMC9834050

[24] Bégaint J, Racapé F, Feltman S and Pushparaja A (2020) Compressai: a pytorch library and evaluation platform for end-to-end compression research. arXiv; *preprint arXiv:2011.03029.* 10.48550/arXiv.2011.03029.

[25] Sonneck J, Zhou Y and Chen J (2024) Mmv_im2im: an open-source microscopy machine vision toolbox for image-to-image transformation. GigaScience 13, giad120. 38280188 10.1093/gigascience/giad120 PMC10821710

[26] Sollmann J and Chen J (2023) AI-based compression applied on brightfield images used for fluorescence prediction. Poster presented at Focus on Microscopy 2023. Available at: https://cdgdep54fthj76t.focusonmicroscopy.org/2023-program-online/?source=pp&event_id=1791&tab=pdf&a_id=3281.

