# Supplementary material for: Deep-learning-based image compression for microscopy images: An empirical study
Source: Biol Imaging. 2024 Dec 20;4:e16. doi: 10.1017/S2633903X24000151 (PMC11704128; doi:10.1017/S2633903X24000151)
Supplement: Zhou et al. supplementary material [file S2633903X24000151sup001.pdf]

Supplementary Material

1 Supplementary Tables

Table S1: Evaluation of the compression ratio and space saving for the different compression methods. First column: compression methods, with the second to the fourth rows as the classic methods and fifth to the last as the deep-learning based methods. The 'Compression ratio' column calculates the ratio between the theoretical image size and the size of the stored bitstream file (the larger the better), while the 'space savings' column is derived from one minus the reciprocal of the previous column. (the larger the better)

**Compression**	**Compression ratio**	**Space saving (%)**
original	1.1236	10.94
JPEGXR	1.3458	24.58
JPEG-2000-LOSSY	**28.5981**	**93.70**
LERC	1.1419	12.36
bmshj2018-factorized-mse-8	15.9426	93.64
bmshj2018-factorized-ms-ssim-8	19.3469	94.82
bmshj2018-hyperprior-mse-8	21.3869	95.07
bmshj2018-hyperprior-ms-ssim-8	23.2744	95.68
mbt2018-mean-mse-8	23.4083	95.50
mbt2018-mean-ms-ssim-8	23.1368	95.65
mbt2018-mse-8	23.746	95.55
mbt2018-ms-ssim-8	22.9054	95.61
cheng2020-anchor-mse-6	**47.2978**	**97.81**
cheng2020-anchor-ms-ssim-6	38.0068	97.35
cheng2020-attn-mse-6	47.0159	97.80
cheng2020-attn-ms-ssim-6	37.3312	97.30

Table S2: Evaluation of prediction accuracy for the downstream label-free task. The test brightfield data are compressed using various AI-based compression technique. The downstream label-free models are also trained on images compressed with the same method used for the test data.

**Compression**	**LPIPS**	**SSIM**	**Correlation**	**PSNR** (dB)
bmshj2018-factorized-mse-8	0.169 ± 0.034	0.662 ± 0.077	0.719 ± 0.097	23.395 ± 1.205
bmshj2018-factorized-ms-ssim-8	0.176 ± 0.035	0.667 ± 0.075	0.714 ± 0.101	23.213 ± 1.272
bmshj2018-hyperprior-mse-8	0.161 ± 0.033	0.692 ± 0.075	0.711 ± 0.100	23.686 ± 1.207
bmshj2018-hyperprior-ms-ssim-8	**0.130** ± 0.029	0.726 ± 0.080	0.725 ± 0.102	**24.484** ± 1.420
mbt2018-mean-mse-8	0.185 ± 0.035	0.621 ± 0.090	0.702 ± 0.100	22.651 ± 1.198
mbt2018-mean-ms-ssim-8	0.182 ± 0.038	0.673 ± 0.077	0.721 ± 0.105	23.117 ± 1.230
mbt2018-mse-8	0.140 ± 0.035	**0.736** ± 0.083	**0.726** ± 0.100	24.358 ± 1.481
mbt2018-ms-ssim-8	0.180 ± 0.035	0.655 ± 0.072	0.719 ± 0.096	23.038 ± 1.119
cheng2020-anchor-mse-6	0.203 ± 0.042	0.625 ± 0.073	0.656 ± 0.108	22.973 ± 1.231
cheng2020-anchor-ms-ssim-6	0.151 ± 0.033	0.681 ± 0.076	0.716 ± 0.100	23.844 ± 1.291
cheng2020-attn-mse-6	0.192 ± 0.036	0.640 ± 0.077	0.658 ± 0.110	22.571 ± 1.254
cheng2020-attn-ms-ssim-6	0.148 ± 0.032	0.688 ± 0.091	0.716 ± 0.100	24.006 ± 1.384

Table S3: Learning setups for the compression model

Learning Setups	2D	3D
Usage	pretrained<br>**no fine-tuning**	Train from scratch
Batch size	16 or 32	2
Epochs	\	50 (MSE) + 50 (SSIM)
Loss function	distortion: MSE + SSIM<br>rate: bitrate entropy loss	distortion: MSE + SSIM<br>rate: bitrate entropy loss
Optimizer	Adam	Adam
Scheduler	ReduceLROnPlateau<br>(patience=20)	ReduceLROnPlateau<br>(patience=20)
Initial learning rate (lr)	1e-4	1e-4
Auxiliary loss learning rate	1e-3	1e-3

Table S4: Learning setups for the downstream label-free model

Learning Setups	2D	3D
Training strategy	Pix2pix Training	Normal Training
model	generator: fnet_2d<br>discriminator: multiscale AvgPool	fnet_3d
Batch size	2	2
Model Selection	EarlyStopping<br>(patience = 50)	EarlyStopping<br>(patience = 50)
Loss function	reconstruction: MSE<br>GAN: BCE	MSE
Optimizer	Adam	Adam
Scheduler	ExponentialLR<br>(gamma = 0.98)	ExponentialLR<br>(gamma = 0.98)
Initial learning rate (lr)	2e-4	1e-3

2 Supplementary Figures

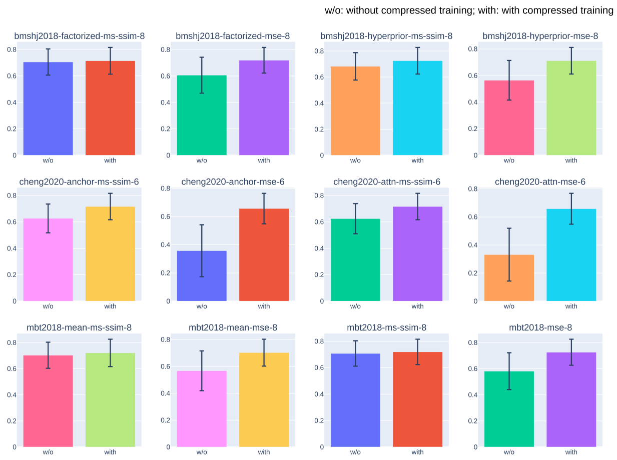

Fig. S1: Performance of the 2D label-free task evaluated using Pearson Correlation. Twelve label-free pix2pix models were trained using images compressed with the same technique applied to the corresponding test data. These results were compared to predictions from models trained on raw, uncompressed images. The models trained with compressed images consistently outperformed those trained with raw images, demonstrating the effectiveness of the compressed training strategy.

3 Supplementary Case Study

3.1 Downstream task: Nuclei Segmentation

Nuclei segmentation is a critical task in biomedical image analysis, providing essential information for various downstream applications such as cell counting, morphology studies, and disease diagnosis. The precision of segmentation results directly impacts the accuracy of these analyses, making it crucial to evaluate the effectiveness of image compression techniques on segmentation performance.

We performed downstream instance segmentation using the StarDist tool [1]. StarDist combines deep learning and geometric modeling to accurately delineate nuclei shapes. For this study, we utilized the DSB2018 2D dataset [2] and the pre-trained checkpoints provided by StarDist, which are specifically trained for segmenting cell nuclei in fluorescence microscopy images. The comparison between predictions using normal and compressed images is illustrated in Fig. S2, and the results on the entire test dataset are listed in Table S5.

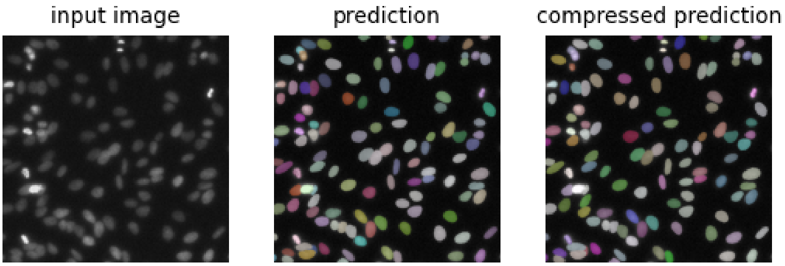

Fig. S2: Comparison of nuclei segmentation predictions from normal fluorescent images and compressed images. From left to right: input normal image, prediction from the normal image, prediction from the compressed image.

Table S5: Performance metrics for nuclei segmentation predictions on DSB2018 test dataset.

Compression	F1 Score	Precision	Accuracy
Original	0.927	0.933	0.864
bmshj2018-hyperprior-8-ms-ssim	0.928	0.936	0.866

For quantitative analysis of nuclei shape and size, we selected three shape metrics: circularity, eccentricity, and perimeter, and calculated size using pixel count. We analyzed these metrics on a selected image containing 122 segmented nuclei. A paired sample t-test was conducted to compare the metrics under normal and compressed conditions. The p-values are listed in Table S6.

Table S6: P-values from paired sample t-tests comparing metrics between normal and compressed images

Metric	Size	Eccentricity	Circularity	Perimeter
p-value	0.2184	0.0442	0.1112	0.9700

The results show that, except for eccentricity, the p-values of all other metrics are greater than 0.05. This indicates that, in general, there is no significant difference in shape and size between the predictions made using the original image and those made using the compressed image.

3.2 Downstream task: Ratiometric Florescent Imaging

Ratiometric fluorescent imaging is a powerful technique used to measure various physiological parameters, such as pH and Calcium concentration, by exploiting the ratio of fluorescence signals from two different fluorophores. This method improves measurement accuracy by normalizing the signal against potential variations in illumination and detector sensitivity.

We followed Munglani *et al.*'s work [3] and reproduced one example in their github repo [1]. Basically, the author measured the pH distribution in the *Arabidopsis thaliana* using pHusion as the ratiometric pH sensor. pHusion consists of the tandem concatenation of enhanced green fluorescent protein (EGFP) as the donor and monomeric red fluorescent protein (mRFP1) as the receptor [4].

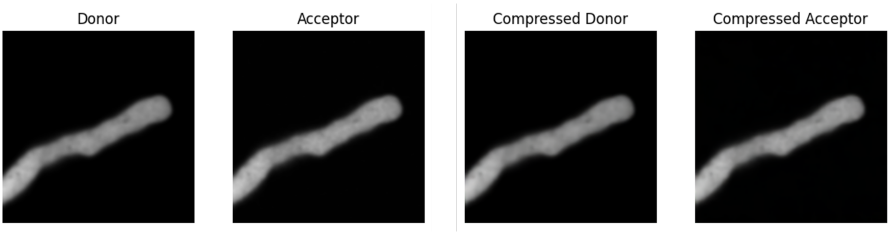

Fig. S3: Comparison before compression and after compression in terms of donor and receptor, respectively

Table S7: Similarity metrics between the raw pH heatmap and the compressed pH heatmap

Metric	SSIM	PSNR	Correlation
Value	0.9831	32.0295	0.9908

[1] https://github.com/gmunglani/fret-ibra

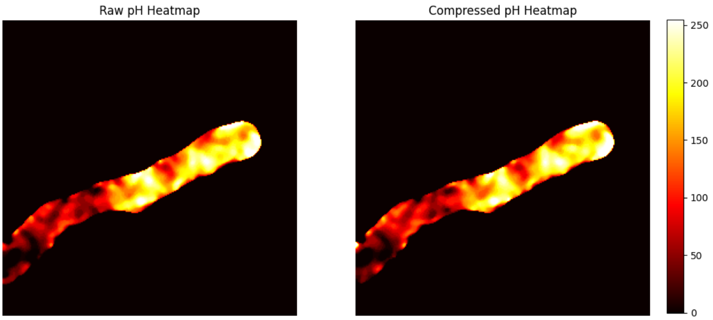

Fig. S4: Comparison between the raw pH heatmap and the compressed pH heatmap.

The result showcases that the pH concentration heatmap almost stays the same, irrespective of whether the donor or the receptor images are compressed or not.

References

[1] Schmidt, U., Weigert, M., Broaddus, C. & Myers, G. *Cell detection with star-convex polygons*, 265–273 (2018).

[2] Goodman, A. *et al.* 2018 data science bowl (2018). URL https://kaggle.com/competitions/data-science-bowl-2018.

[3] Munglani, G., Vogler, H. & Grossniklaus, U. Fast and flexible processing of large fret image stacks using the fret-ibra toolkit. *PLoS Computational Biology* **18** (4), e1009242 (2022) .

[4] Gjetting, S. K. *et al.* Live imaging of intra-and extracellular ph in plants using phusion, a novel genetically encoded biosensor. *Journal of experimental botany* **63** (8), 3207–3218 (2012) .
